# Biodegradation of propiconazole by newly isolated *Burkholderia* sp. strain BBK_9

**DOI:** 10.1007/s13205-016-0429-3

**Published:** 2016-05-11

**Authors:** Praveen Satapute, Basappa Kaliwal

**Affiliations:** 1 Department of Microbiology and Biotechnology, Karnatak University, Dharwad, Karnataka India; 2 Department of Microbiology and Biotechnology, Davangere University, Davangere, Karnataka India

**Keywords:** Propiconazole, Biodegradation, *Burkholderia* sp. metabolites, Plasmid curing

## Abstract

The isolation of propiconazole (PCZ) degrading bacterium BBK_9 strain was done from paddy soil, and it was identified as *Burkholderia* sp. based on the morphological characteristics and biochemical properties combined with 16S rRNA gene sequencing analysis. It has been seen that the factors such as temperature and pH influence the biodegradation process. The role of plasmid was studied in the degradation process by plasmid curing method. The PCZ acts as the sole carbon source and as energy substrate which can be utilized by the strain for its growth in Mineral salt medium and degraded 8.89 µg ml^−1^ of PCZ at 30 °C and pH 7 within 4 days. During the bioconversion process of PCZ, three metabolite were formed such as 1-(2,4-dichlorophenyl)-2-(1*H*-1,2,4-triazol-1-yl) ethanone, 1-[2-(4-chlorophenyl) ethyl]-1*H*-1,2,4-triazole and 1-ethyl-1*H*-1,2,4-triazole. The LD50 value of BBK_9 strain was determined with acridine orange which resulted in 40 µg ml^−1^ at cell density of 0.243 at 660 nm. Furthermore, plasmid curing was done using LD50 concentration and from that three plasmids got cured in the sixth generation. It was found that, cured strain was able to degrade 7.37 µg ml^−1^ of PCZ, indicating the plasmid encoded gene were not responsible for the PCZ degradation. On the source of these outcomes, strain BBK_9 can be used as potential strain for bioremediation of contaminated sites.

## Introduction

The economy of India is largely depend on the agricultural production, due to the implementation of advanced technologies in the form of bio fertilizers, chemical fertilizers and different forms of pesticides have made possible to increase the quality and quantity of field products (Ramudu et al. 2011). Moreover, attention on the pesticides has increased during the recent times due to their versatile nature and various modes of action. Currently, triazole fungicides are the extensively used among the all fungicides for the protection of standing crops against plant pathogens. Whereas, the toxicity and non-target effect of azole fungicides are vastly under represented.

Propiconazole (PCZ) is a triazole foliar fungicide, which is used in the agriculture. The yearly intake of this fungicide is about 7373 g a.i. ha^−1^. The degradation of pesticides by microorganisms is gaining the extensive attention in the agricultural field and environmental microbiology. Interestingly, only few literatures are there which are dealing with the fungicide degradation by bacteria and in the previous years, many experiments have suggested that bacterial community are effective degraders of many different classes of fungicides: vinclozolin (Lee et al. 2008), tubeconazole (Nicole et al. 2009), captan (Megadi et al. 2010), benzimidazole (Cycon et al. 2011) and thiram (Sherif et al. 2011) and is necessary since PCZ biodegradation was not thoroughly investigated with the exception of Sarkar et al. (2009). Moreover, previously available reports are mentioned that PCZ utilization by microorganisms is not possible because of its ability to strongly adsorb with the soil’s organic matter (Kloskowski et al. 1987; Ekler 1988; Woo et al. 2010). Similarly, laboratory scale degradation has been conducted in the different soils using commercial grade of PCZ alone or the mixture with benazone, dichlorprop and 2-methyl-4-chlorophenoxyacetic acid. It was revealed that the highest persistence of PCZ was found in agricultural soil since its traces were seen even after 84 days duration of experiment. (Thorstensen and Lode 2001). As per the technical information bulletin for PCZ, the half life ranges from low 96 days in a sandy loam to 575 days in a silt loam. Similarly, PCZ degradation by photolytic method in water is as long as 249 days. Thus, there is need of effective and cost friendly techniques which can degrade the PCZ from contaminant sites.

The isolate grown in respective medium is necessary to produce important metabolites which are precursors of degradation and it is also important to know that, potent microorganisms are likely to yield important new information on how the isolated bacteria is capable of degrading the toxic pesticide that prevails in the agricultural soil. Therefore, this study was under taken to isolate the potent microorganism which will prove their capability to metabolize PCZ under the liquid medium in given optimal conditions. Also, the function of plasmid and ability of cured strain in degrading the PCZ was investigated.

## Materials and methods

### Chemicals and reagents

Propiconazole was obtained from Nagarjuna Agrichem Co., Ltd (Srikakulam, India), ethyl acetate and acetonitrile of HPLC grade were purchased from the HiMedia and all other chemicals, and reagents were used for this experimental study are highest analytical grade.

### Soil samples and media

For this study, soil was collected from the uppermost layer (0–10 cm) of paddy field with the pesticide application situated in the Koppal district of Karnataka, India. The mineral salt media (MSM) (Seubert 1990) was used for this study.

### Isolation of PCZ utilizing bacteria

The samples of soil which were collected from the different places were aseptically brought to the laboratory, 1 g of sieved soil was added in 100 ml of MSM and the flasks were placed in the incubator shaker at 30 °C in 100 rpm for 1 h. Further, 10 ml of soil suspension was inoculated to 100 ml of sterile MSM broth containing 1 µg ml^−1^ of PCZ as the only carbon source and energy substrate, incubation of all the flasks was done for 7 days at 30 °C in 120 rpm. After the incubation period the serial dilution of media was done up to 10^−7^ and appropriate dilutions (10^−5^, 10^−6^) were inoculated in nutrient agar (NA) medium containing PCZ (1 µg ml^−1^) and all the colonies that were appeared on the NA were purified by quadrant streak method and stored at 4 °C.

### Screening and acclimatization

Several different colonies were isolated, all colonies that were appeared on the NA were examined for their PCZ utilizing capacity depending upon the colony count on mineral salt agar medium (MSAM) in different concentration of PCZ (2 and 10 µg/L). Further, highly potent strain was acclimatized for the better development and utilization of PCZ degrading bacterium.

### Identification of PCZ utilizing bacteria

The potent PCZ utilizing soil isolate was identified as per the morphological, Gram’s characteristics and biochemical tests. Further, 16S rRNA sequencing was done at Xcelris genomics, Ahmedabad, India. Isolation of bacterial genomic DNA was done using xcelgen genomic DNA isolation kit and bacterial 16S rRNA gene fragment was amplified by PCR (Eppendorf Thermal Cycler) from genomic DNA using 16S gene universal primers: 8F and 1492R. Further, amplified PCR product was purified using xcelgen gel extraction kit. After determination of purified DNA, sequencing was done using automated DNA sequencing on ABI 3730xl Genetic Analyzer (Applied Biosystems, USA). Sequencing was done using Big Dye^®^ Terminator v3.1 Cycle. Further, to perform the pairwise/multiple sequence alignment, the CLUSTALW tool was used and phylogenetic tree was constructed by the neighbor-joining method using bootstrap test (1200 replicates) (Saitou and Nei 1987), the evolutionary distance was computed using the MEGA 7 package by Tamura and Nei (1993) method and sequence of organism was deposited in the gene bank with the accession number LC099979.

### Utilization of PCZ by *Burkholderia* sp. BBK_9 strain

Propiconazole resistant strain was pre cultured in nutrient broth at 30 °C in rotary shaker, and 3 × 10^6^ cell ml^−1^ from the same media was inoculated in 100 ml of liquid MSM containing 10 µg ml^−1^ of PCZ which acts as sole carbon source and growth substrate, then incubated at 30 °C in rotary shaker with 120 rpm. The cultures were periodically collected and measured the OD at 600 nm for cell growth, and bacterial suspension of all samples was centrifuged at 10,000*g* for 15 min at 4 °C. Further, culture filtrates were extracted with the ethyl acetate in rotor flash evaporator (Buchirotavapor R 210) and the residues were dissolved in the acetonitrile (HPLC grade) to examine the utilization of PCZ by UV spectrophotometer (Hitachi U 2900) at 220 nm. All the tests were performed in three replicates. Percentage of PCZ degradation was calculated by formula which was previously mentioned by the Rokade and Mali (2014).

$$\% {\text{ of propiconazole degradation}} = {\text{Ab}} - {\text{Aa}}/{\text{Ab}} \times 100$$

where, Ab is initial percentage of PCZ in MSM (before degradation) and Aa is percentage of PCZ after 4 days.

### Extraction of metabolites by preparative HPLC

The extraction and purification of metabolites was done by the preparative HPLC on an Agilent Zorbax SB-C18 preparative column (250 × 9.4, 5 µm). For the preparation of the three fractions, chromatographic peaks were collected at the exit of the detector into three different pools. Subsequently, the compounds or the pools were concentrated by removing/evaporating the solvent.

### Biodegradation analysis

To quantify the degradation rate of PCZ by BBK_9 strain, the HPLC method was followed with little modification and was described by Bromilow et al. (1999). Briefly, 20 µl of test sample was injected into HPLC Agilent 1260 fitted with quaternary pump, auto sampler and variable wavelength UV detector with C 18 column (diameter 150 × 4.6 mm) having particle size 5 micron and elution of samples were done at 1.5 min ml^−1^ with the mobile phase methanol + water (70:30). Mass spectrum (MS) was used for the analysis of metabolites (Shimadzu). One microliter of each sample was examined at MS by conditions: the transfer line temperature was 240 °C, with 230 °C ion trap and 120 °C manifold temperature. Full-scan (60–550 *m/z*) EI (auto mode) with 10 mA filament current was used for MS analysis from 5–31 min, which gave 2.7 scans/s. Target automatic gain control was 15,000, and the multiplier voltage was 1450 V. The mass scan range at 1000 in the electron ionization mode (EI).

### Effect of temperature and pH on biodegradation of PCZ

The optimal condition required for degrading PCZ was tested in MSM amended with the PCZ (10 μg ml^−1^), the pH tolerance of the isolate necessary for degrading PCZ was examined at pH values of among 5.0–9.0 and pH of MSM was adjusted using the 1N NaOH and 1N HCL. Similarly, optimum temperature conditions were studied ranging between 20 and 60 °C. The degradation percentage was monitored by the UV spectrophotometer (Hitachi U 2900) at 220 nm. All trials were performed in three replicates and in flasks which were not inoculated served as control.

### LD 50 value determination and plasmid curing of isolate BBK_9

#### LD 50 value determination

The bacterial isolate BBK_9 was grown in nutrient broth for 18 h and inoculated (0.1 ml) into nutrient broth tubes (5 ml) with different concentrations of acridine orange (5–50 µg ml^−1^). Control tube without acridine orange was also maintained. The culture was grown for 24 h at 37 °C and the biomass was measured at 660 nm. The OD values of treated samples were compared with that of control and LD50 concentration was determined.

#### Plasmid curing

Plasmid curing of isolate BBK_9 strain was determined by the LD50 concentration was used to treat the bacterial isolate for five to six generations. The inoculation of test organism was done in 25 ml of nutrient broth which was autoclaved and incubated at 37 °C for 18 h. Five tubes of nutrient broth containing LD50 concentrations of acridine orange were prepared and 18 h old test organism was inoculated (1 %) in tube no. 1. The control tubes without acridine orange were also prepared and inoculated, as mentioned earlier, the incubation was done at 37 °C for 24 h. 1 % culture from tube no. 1 was inoculated to tube no. 2 and incubated further for 24 h. This serial inoculation was continued till plasmid curing was attained. The presence/absence of plasmid was observed by running it on 1 % agarose gel by isolating the plasmid at every generation. Meanwhile, the plasmid cured BBK_9 strain was examined for its capability for degrading PCZ.

### Statistical analyses

All Experiments were carried in a three independent replicates. Analysis of data was done by one way ANOVA variance SPSS version 20.0 software and data were denoted as means ± standard errors. The difference among the each means were located using Duncan’s test (*P* < 0.05).

## Results

### Isolation, screening and characterization of PCZ utilizing bacteria

Total of 11 strains (BBK_1-11) were isolated from the paddy cultivated soil. Quadrant streak method was utilized for purifying all strains on nutrient agar plates containing PCZ (1 µg ml^−1^). Further, strains were screened for their ability to utilize PCZ in different concentrations (2–10 µg ml^−1^). As a result, BBK_9 strain was found most promising candidate for the utilizing of PCZ with a substantial growth in the all concentration. For the better enhancement of degradation process BBK_9 strain was acclimatized in MSM amended with PCZ (10 µg ml^−1^).

### Identification of BBK_9 strain

**Fig. 1:**
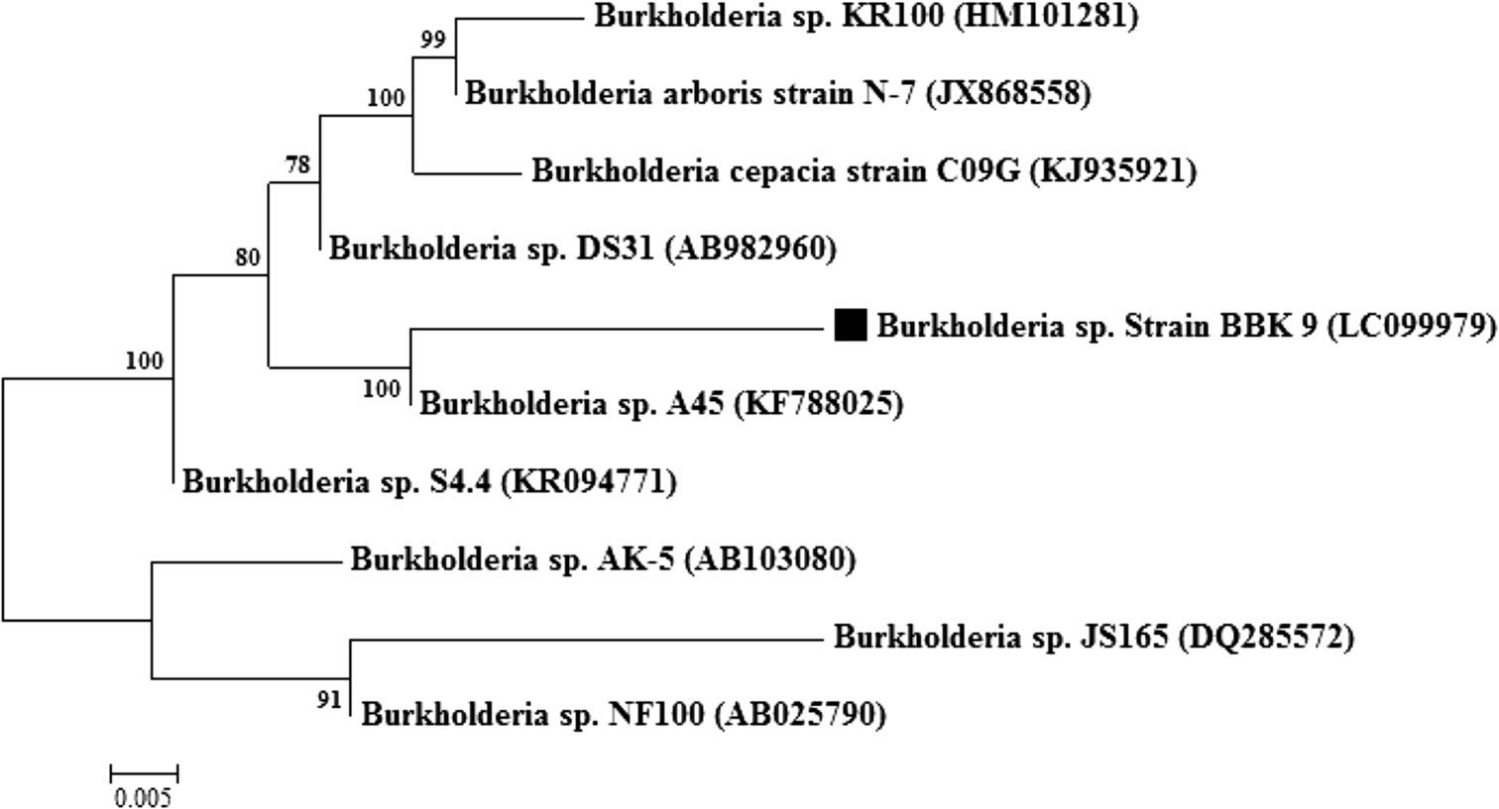
Phylogenetic tree analysis of PCZ degrading strain, constructed through neighbor-joining method with bootstrap values as percentage at the nodes

The BBK_9 strain was found Gram negative, non-motile, aerobic, rod shape. Biochemically it was found catalase and urease positive. The blast analysis of 16S rRNA sequence of isolate BBK_9 strain reveals closer resemblance with the *Burkholderia* sp. A45 strain (Genebank accession number KF788025). The phylogenetic tree shows the evidence for identifying the isolate which is represented in Fig. 1. The phylogenetic tree of BBK_9 was constructed with those of the soil isolates, with 78–100 % homology to those of species that are known to be potent degraders of pesticides and heavy metals.

### Utilization of PCZ by *Burkholderia* sp. BBK_9 strain

**Fig. 2:**
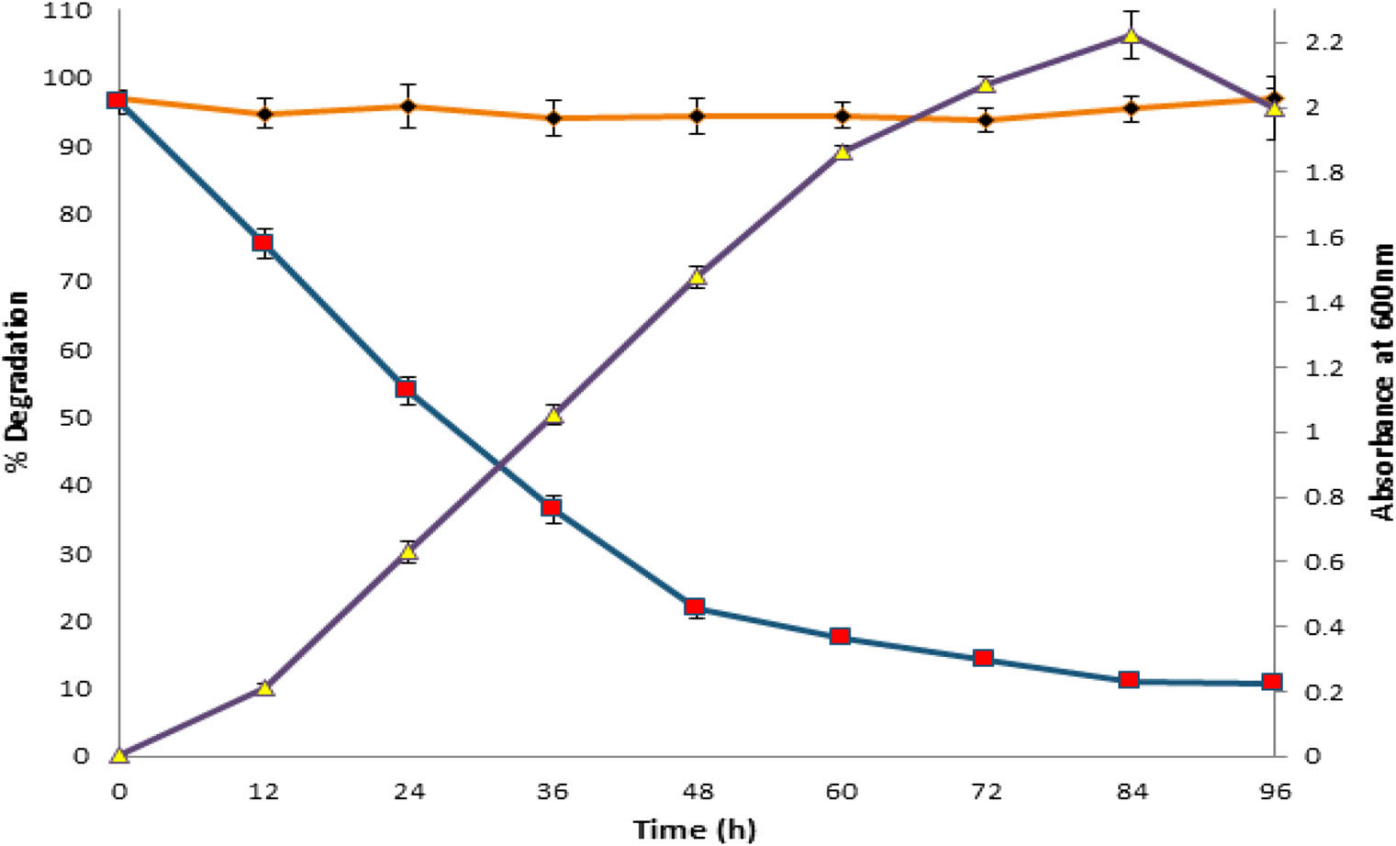
Time course degradation of PCZ. Degradation rate (–■–) vs. bacterial cell growth of strain BBK_9 regarding to the alteration in turbidity (–▲–) and uninoculated controls (–●–) in the mineral salt media. *Error bars* represents mean ± standard error (SE) of three replicates

The bacterium *Burkholderia* sp. BBK_9 strain was tested for its PCZ degrading capability and the biodegradation was achieved up to 8.89 µg ml^−1^ (88.87 %) after the incubation of 4 days. Bacterial growth and degradation was measured at 600 and 220 nm, respectively. In the meantime, no abiotic loss was observed in the control flasks and its proof is given in the Fig. 2.

### Effect of temperature and pH on PCZ degradation

**Fig. 3:**
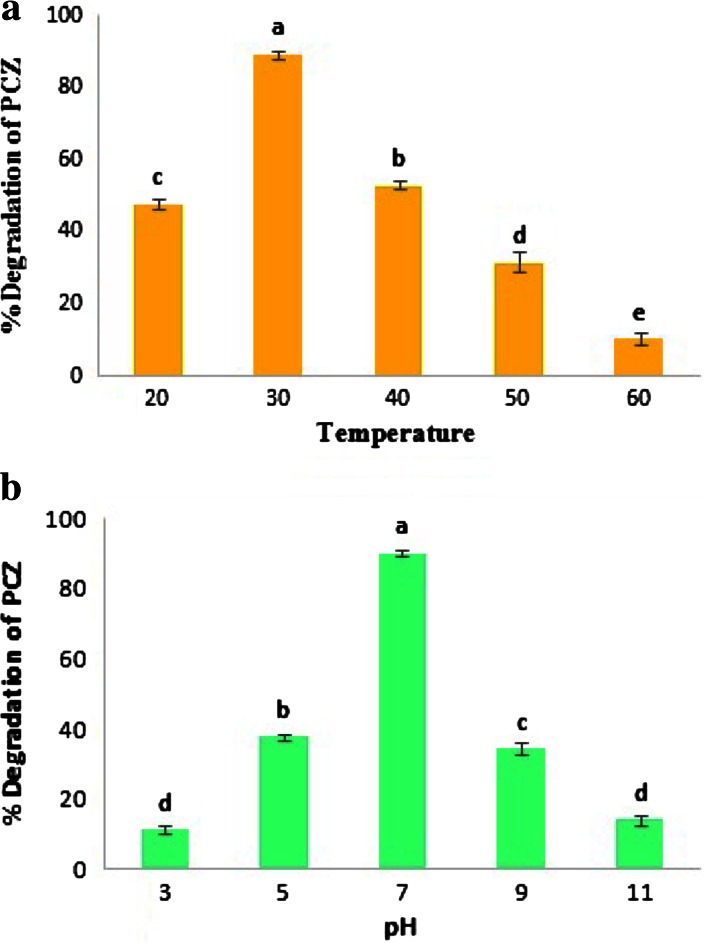
**a** Effect of temperature on degradation rate of PCZ. **b** Impact of pH on degradation of PCZ. All the values are mean ± SE for each experiment are significantly different (*P* ≤ 0.05) from each other according to Duncan’s test

It was evident that, the upper and lower temperatures are responsible to affect the degradation rate of PCZ. The maximum degradation was achieved at optimum condition 30 °C with degradation up to 89.31 %. However, the degradation rate was decreased up to 47.73 % at 20 °C and 10.55 % at 60 °C (Fig. 3a). Likewise, the degradation rate of PCZ also gets affected by higher and lower pH values, pH 7 (90.31 %) was found optimum condition and least degradation was obtained at pH 3 (11.19 %) and pH 11 (13.94 %) (Fig. 3b).

### Biodegradation analysis

**Fig. 4:**
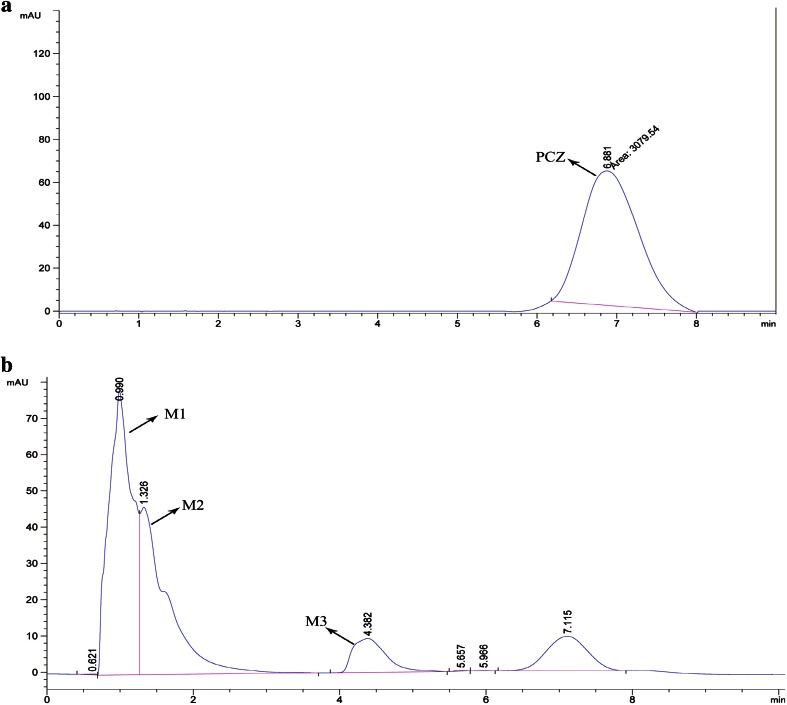
**a** HPLC peak of standard propiconazole. **b** HPLC peak of 4 days incubated cell free culture filtrate showing presence of metabolites

**Fig. 5:**
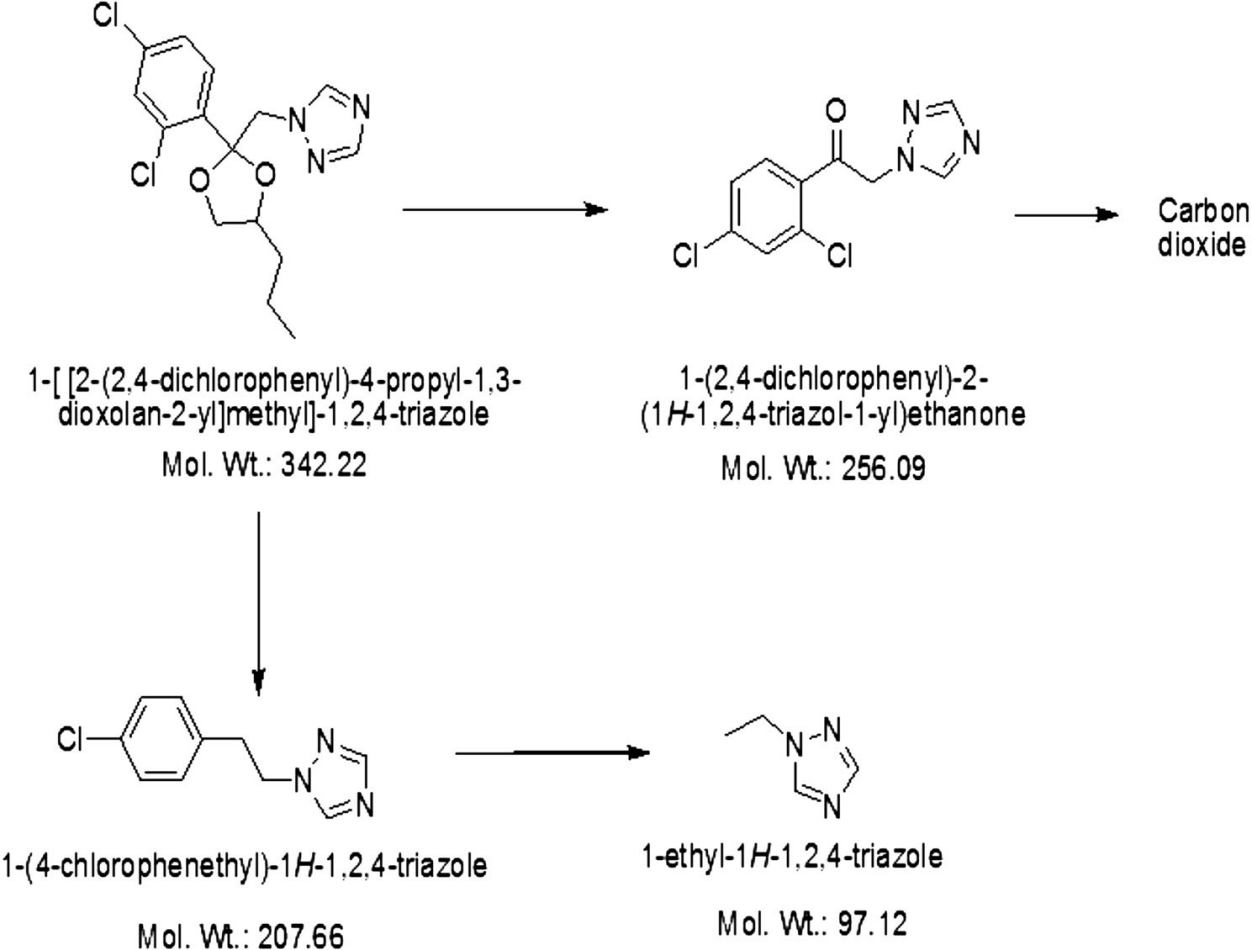
Proposed degradation pathway of propiconazole by *Burkholderia* sp. BBK_9 strain

The standard PCZ was eluted at the retention time 6.8 min (Fig. 4a) with single peak and also in D0 (before inoculation of bacterial suspension) and mass of PCZ was determined by the mass spectra at *m/z* 342.22. However, culture filtrate extracted on the first, second, third and fourth days showed the considerable decrease in the PCZ peak with the formation of other metabolite peaks, which confirms degradation of PCZ. It is necessary to consider that, the culture filtrates extracted in different days showed the two important stable metabolites (M1 and M2) having retention time of 0.9 and 1.3 min, respectively, and also other metabolites were mineralized during entire degradation process. Further during fourth day at 4.3 min other compound (M3) was detected (Fig. 4b). Metabolites were extracted by preparative HPLC and identified by fragmentation pattern and *m/z* values such as 1-(2,4-dichlorophenyl)-2-(1*H*-1,2,4-triazol-1-yl) ethanone (*m/z* 256) (M1), 1-[2-(4-chlorophenyl) ethyl]-1*H*-1,2,4-triazole (*m/z* 207) (M2) and 1-ethyl-1*H*-1,2,4-triazole (*m/z* 98), respectively. The structural confirmations of all metabolite were assigned from the fragmentation and *m/z* values procured. The proposed pathway for the PCZ degradation is showed in Fig. 5.

### LD50 value determination and plasmid curing of isolate BBK_9

**Fig. 6:**
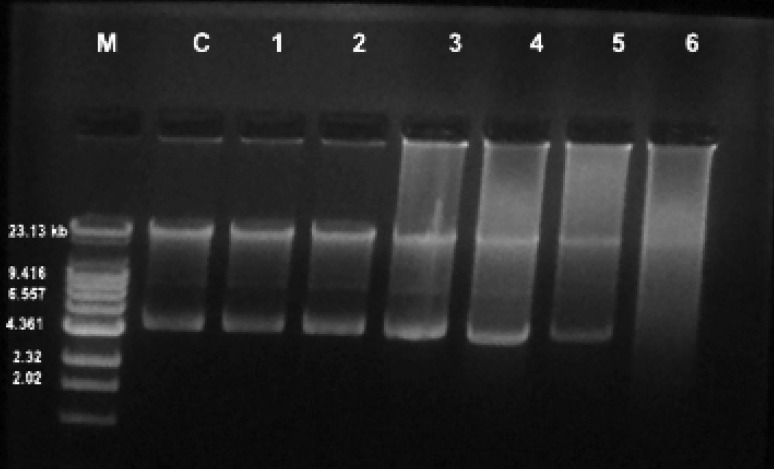
The effect of curing agent acridine orange on BBK_9 strain; *lane1* Marker, *lane2* control, *lane 3* and *4* showing the presence of plasmids in the first and second generation, *lane 5*, *6* and *7* plasmid curing with hampered plasmid at the third, fourth and fifth generation, *lane 8* complete curing of plasmid at sixth generation

**Fig. 7:**
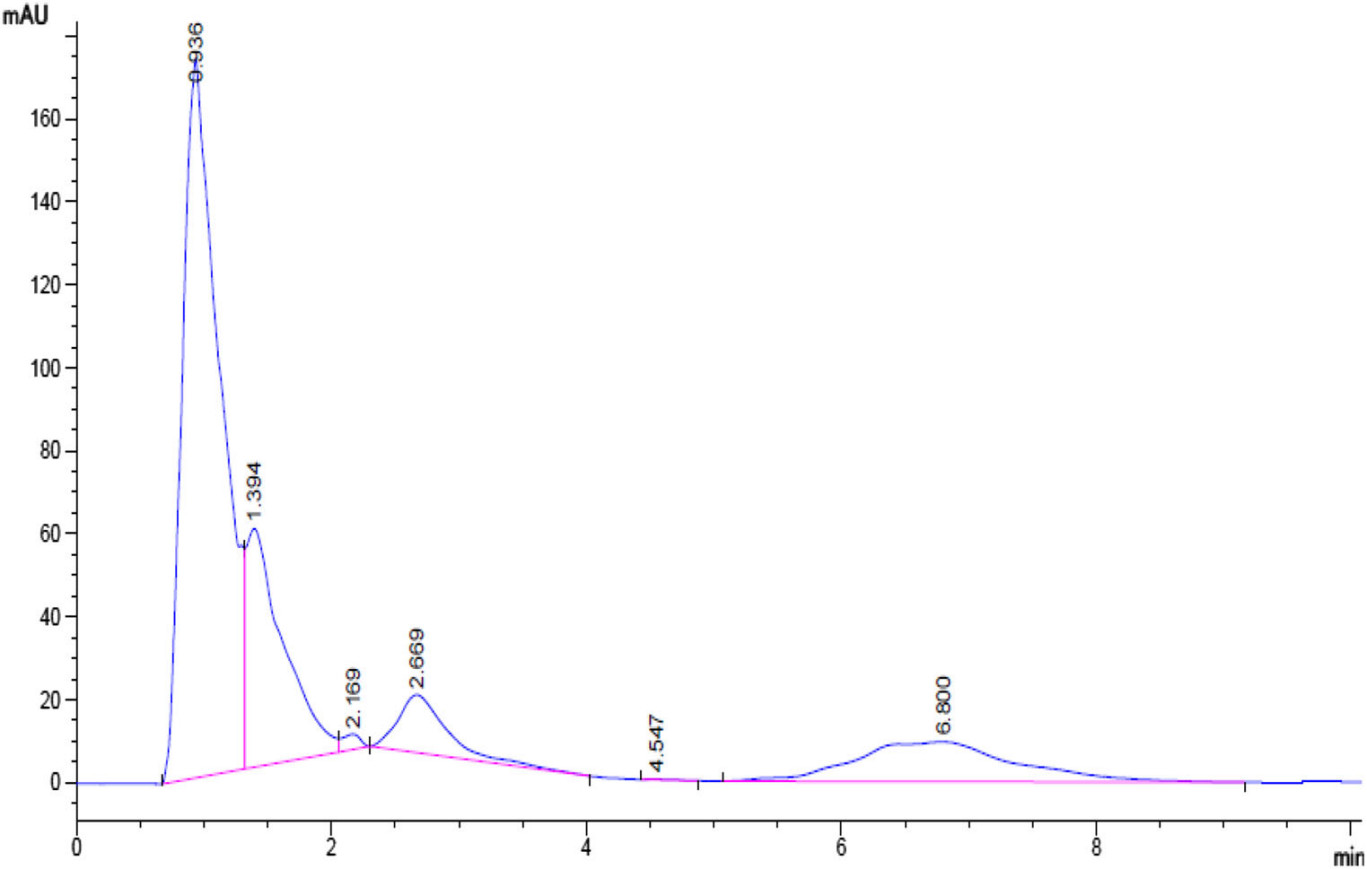
HPLC chromatogram of propiconazole extracted through culture filtrate of plasmid cured strain showing the degradation up to 7.37 µg ml^−1^

LD50 value was determined by amending the acridine orange in media inoculated with BBK_9 strain. LD50 value of BBK_9 strain was observed to be 40 µg ml^−1^ at cell density of 0.243 at 660 nm, which is 0.443 half the density of the control. The plasmid curing of BBK_9 strain was performed by LD50 concentration up to sixth generation, the presence or absence of plasmid at every generation was analyzed by isolating it. It was found that, the three plasmids were present at approximately 23.13, 9.14 and 4.36 kb, respectively, plasmids were hampered after the second generation. It indicates that, the curing process has taken place and the complete plasmid curing was observed at sixth generation (Fig. 6). Furthermore, plasmid cured strain was examined for its PCZ degradation capacity, which showed that, the substantial growth of plasmid cured organism in MSM was attained with degradation of PCZ up to 7.37 µg ml^−1^ at the initial concentration of 10 µg ml^−1^(Fig. 7).

## Discussion

In this study, we have isolated most impending candidate for degrading the PCZ from the fungicide contaminated paddy soil. It was evident that, the population of *Burkholderia* sp. was observed to be predominant in the paddy soil and the bacterium *Burkholderia* sp. BBK_9 strain was evidenced for its ability to utilize PCZ as sole carbon and growth substrate in liquid medium resulting 8.89 µg ml^−1^ (88.87 %) of degradation under the optimal conditions pH 7 and 30 °C. The preparative HPLC method came across as the most useful method for extracting and identifying the metabolites. Furthermore, culture filtrate yielded three precursors of PCZ degradation namely, 1-(2, 4-dichlorophenyl)-2-(1*H*-1, 2, 4-triazol-1-yl) ethanone (M1), 1-[2-(4-chlorophenyl) ethyl]-1*H*-1, 2, 4-triazole (M2) and 1-ethyl-1*H*-1,2,4-triazole (M3). Based on the fragmentation pattern M1 was identified with degradation of dioxolane ring from the PCZ representing the degradation product with molecular weight of 256. Simultaneously, M2 resulted from the loss of PCZ dioxolane ring and elimination one chlorine atom from the PCZ with molecular weight of 207. Metabolite M3 is identified by the loss of 1-chloro-4-methylbenzene with *m/z* value of 98. This experimental study would throw the light on plasmid role in the degradation. Moreover, plasmid cured strain was also an able contender in degrading the PCZ and it was clear that plasmid encoded genes does not play any role in the degradation process.

Isolation of pesticide resistance bacteria from the contaminated soil is the initial stage to understand the microbial population and to evaluate their tolerant capacity to the particular environment. Previously, Goda et al. (2010) isolated the five malathion degrading bacteria from Egyptian soil, among the all isolates *Pseudomonas putida* strains were chosen to conduct the degradation study due to their high efficacy and the important of this strain is revealed by repeated enrichment isolation method. The another research from united kingdom reveals the potential of isolation and screening of perithroid degrading bacteria which is isolated from the soil and in this study the potent bacterial strains were screened based on colony morphology which is having largest size (Grant et al. 2002). *Paracoccus* sp. strain TRP was able degrader of chlorpyrifos (CP) and 3,5,6-trichloro-pyridinol (TCP) and it was the supreme candidate for the degradation of both the compounds in higher concentration than the other isolated strains and the screening of all strains were evaluated by CP and TCP utilizing capacity (Xu et al. 2008). This suggesting the screening for the potent strain in the biodegradation study is most important parameter.

In general, *Burkholoderia* is a genus of proteobacteria which is having impressive applications in the field of agricultural, as a potent agent in the process of biodegradation, biocontrol, and plant growth-promoting rhizobacteria (Estrada-de Los Santos et al. 2013) and also reported for its potentiality for degrading pesticides (Hong et al. 2007; Kim and Ahn 2008; Pimmata et al. 2013). Degradation of many pesticides by microorganisms under the in vitro conditions is mainly temperature and pH dependent. In support to this study, Kim et al. (2002) have given the evidence that temperature will be the major key to degradation of PCZ. Standardization of optimized parameters has significant influence over any biodegradation process and also reported that acidic and alkaline conditions were not suitable for degradation experiments due to their ability in limiting the growth of bacteria (Phugare et al. 2012). Recently, Talwar et al. (2014) has reported the studies on optimized conditions in degrading the quinalphos. Interestingly, PCZ degradation is assisted by UV in the presence of titanium dioxide (TiO_2_) as a catalyst in aqueous solution under optimum conditions like pH (6.5), intensity of light (30 Wm^−2^) and constant temperature (25 ± 1 °C) during the period of experiment (Kaur et al. 2015). The physical parameters (pH and temperature) will have greater influence on the degradation of PCZ.

A limited information is available for microbial degradation of PCZ, in support to our current study, Sarkar et al. (2009) suggesting, *P. putida* is the most effective candidate for the utilization of PCZ under the liquid medium and also carbon source like glucose will enhance the process of degradation. Although, none of the PCZ metabolites were not reported. More importantly, metabolic pathway of PCZ by *Burkholderia* sp. BBK_9 is reported for the first of its kind. The white rots, brown rots and moulds were investigated by wood matrix method for their degradation capacity of carbon based preservatives, and it was achieved up to 75 % of degradation applied at 1.39 kg m^−3^ (Woo et al. 2010). Above mentioned two literatures suggests that carbon sources will be the major key factor in degrading PCZ under different method of degradation process. It indicates that influence of energy source will have the impact on degradation mechanism.

Correspondingly, Kim et al. (2002) studied the fate of PCZ in paddy soil lysimeter and possible metabolites. Namely, 1-[[2(2,4-dichlorophenyl)-2-(1,2,4-triazole-1-yl) ketone (DP1), 1-(2,4-dichlorophenyl)-2-(1,2,4-triazole-1-yl) ethanol (DP2) and 1[[2(2,4-dichlorophenyl)-4-hydroxypropyl-1,3-dioxolane-2-yl]methyl]1*H*-1,2,4triazole. Further, carbon dioxide and bound residue were found to be metabolites of the PCZ. The metabolite DP2 was found similar in our investigation, this supports the present experimental results. Importantly, the dioxolane ring degradation from the PCZ is the initial step in degradation process. Vialaton et al. (2001) studied the photolysis of PCZ in pure water, in water containing humic substances and in natural water, it was revealed that PCZ was photo degraded and the dissolution of PCZ was found 25 % faster in natural water then the pure water. Similarly, the fate of PCZ degradation in canal water was tested under the laboratory conditions; which showed the dissipation of PCZ up to 80 % (Chauhan et al. 2008). Worryingly, the triazole fungicides are used more than the other group of fungicides but the studies regarding microbial degradation have been vastly under represented. Additionally, PCZ is major triazole agent, it is known to be highly persistent in soil and it will prevent the microorganisms from existing in the soil with long term effect (Yen et al. 2009). Importantly, the fungicides were never classified and characterized according to their mode of action, it indicates the problems for the assessing the non-target effect. Conversely, for the protection of biological functions and soil fertility, selection of fungicides is very important to prevent the non-target effects (Yang et al. 2011).

Previously, five triazole fungicides were investigated for their degradation ability in peaches field by photo degradation method. Expect PCZ, residual levels of cyproconazole, penconazole, tebuconazole and hexaconazole were found higher the recommended limits at preharvest time. Further, it is also reported that PCZ is highly stable and it will decrease in concentration during the plant growth by diluting effect, it indicates that PCZ is the stable among the other triazoles (Angioni et al. 2003). Additionally, triazole fungicides are globally used for the commercial purpose and most of them are known to be persistent in soil for longer duration and the studies over field have suggests that traces of triazoles will have impact on the soil fertility along with crop yielding (Bromilow et al. 1999). Triazole fungicide often shares a same mode of actions (Li et al. 2013; Satapute and Kaliwal 2015). Hence, *Burkholderia* sp. BBK_9 strain may also be useful in degrading the other triazole pesticides.

Plasmid cured strain was well distinguished for gaining of the resistance over PCZ in current study; it indicates that chromosomal genes are directly responsible for degrading the PCZ by BBK_9 strain. However, only few bacteria were isolated which carry an extra chromosomal DNA. The plasmid cured *Pseudomonas aeruginosa* MCMB-427 strain was found unable in degrading the dimethoate in liquid media. Suggesting, plasmid (designated as pDMD427) has direct involvement in the degradation (Deshpande et al. 2001). Interestingly, other plasmid cured *Pseudomonas aeruginosa* strain which is heavy metal resistant strain found potent candidate to utilize cadmium, chromium and lead indicating that it contains chromosomal genes in degradation mechanism (Raja and Selvam 2009). In case of degradation of fenitrothion, *Burkholderia* sp. strain NF100 is the supreme agent and it was investigated that degradation was encoded by the involvement of plasmids designated as pNF1 and pNF2 (Hayatsu et al. 2000). The slow rate of imidacloprid degradation was found by plasmid cured *Brevundimonas* sp. MJ15 strain. Interestingly, the degradation was archived up to 50 % lesser than the uncured strain. Suggesting, both chromosomal and extra chromosomal genes may involve in degradation process of imidacloprid (Shetti and Kaliwal 2012). Whereas, plasmid of *Pseudomonas aeruginosa* contains the plasmid encoded genes which take part in the degradation mechanism of methomyl (Kulkarni and Kaliwal 2014). Thus, the use of *Burkholderia* sp. BBK_9 strain for biodegradation of hazardous chemicals to make toxic-free environment is possible.

## Conclusion

In this experimental study, the isolation of a potent *Burkholderia* sp. strain BBK_9 was done from paddy soil and also its ability to utilize PCZ was assessed. The isolate was able to transform PCZ into three important metabolites. These experimental results suggested that isolate degraded PCZ up to 8.89 µg ml^−1^ (89 %). Along with that the strain preserved its ability to utilize PCZ at 30 °C and pH 7. Plasmid cured cells were also a healthy competitor which can degrade the PCZ. Indicating additional studies to advance more widen view over PCZ degrading enzymes and gene from *Burkholderia* sp. BBK_9 strain. Thus, results of this study showed that the *Burkholderia* sp. strain BBK_9 can play a role as significant biological candidate for cleaning up of the contaminated pollutants.
